# Understanding the inverse care law: a register and survey-based study of patient deprivation and burnout in general practice

**DOI:** 10.1186/s12939-014-0121-3

**Published:** 2014-12-12

**Authors:** Anette Fischer Pedersen, Peter Vedsted

**Affiliations:** Research Unit for General Practice and Research Centre for Cancer Diagnosis in Primary Care (CaP), Health, Aarhus University, Aarhus, Denmark

**Keywords:** Burnout, General practice, Inverse care law, Medical care supply, Patient deprivation

## Abstract

**Introduction:**

According to the inverse care law, there is a mismatch between patients’ medical needs and medical care supply. As an example, the number of doctors is often lower in areas with high deprivation compared to areas with no deprivation, and doctors with a deprived patient population may experience a high work pressure, have insufficient time for comprehensive tasks and be at higher risk for developing burnout. The mechanisms responsible for the inverse care law might be mutually reinforcing, but we know very little about this process. In this study, the association between patient deprivation and burnout in the general practitioners (GPs) was examined.

**Methods:**

Active GPs in the Central Denmark Region were invited to participate in a survey on job satisfaction and burnout and 601 GPs returned the questionnaire (72%). The Danish Regions provided information about which persons were registered with each practice, and information concerning socioeconomic characteristics for each patient on the list was obtained from Statistics Denmark. A composite deprivation index was also used.

**Results:**

There was significantly more burnout among GPs in the highest quartile of the deprivation index compared to GPs in the lowest quartile (OR: 1.91; 95% CI: 1.06-3.44; p-value: 0.032). Among the eight variables included in the deprivation index, a high share of patients on social benefits was most strongly associated with burnout (OR: 2.62; 95% CI: 1.45-4.71; p-value: 0.001).

**Conclusions:**

A higher propensity of GP burnout was found among GPs with a high share of deprived patients on their lists compared to GPs with a low share of deprived patients. This applied in particular to patients on social benefits. This indicates that beside lower supply of GPs in deprived areas, people in these areas may also be served by GPs who are in higher risk of burnout and not performing optimally.

## Introduction

According to the inverse care law, the availability of medical care tends to vary inversely with the need for it in the population served [1,2]. For instance, in primary care, deprived patients have been shown to have reduced access to scheduled encounters and to spend less time with the general practitioner (GP) during consultations than more affluent patients [3-5]. This is true despite the fact that patients from deprived areas often present with a higher number of problems in the clinical encounter than patients from more affluent areas [3]. Patients from poor areas have been shown to perceive the GP as less patient and less caring than patients from more wealthy areas [6] and a negative correlation between patient deprivation and patient-rated enablement in encounters for psychosocial problems has been reported [3].

These factors reflect double trouble for people in deprived areas as they often have higher levels of physical and mental multimorbidity and problems of psychosocial nature than people in less deprived areas.

Even though GPs tend to be inversely distributed with fewer in deprived areas, the clinical activities have been shown to be higher in deprived areas compared to more affluent [7]. This could mean that GPs working in such areas would be exposed to more stressful conditions that could lead to lower performance and job satisfaction. Eventually, the GP may move from the area or stop practicing contributing to the inverse care law. Although it is universally accepted among GPs and researchers that the work is more stressful in areas of social deprivation [8], the actual association between deprivation and perceived workload is not well-investigated.

Burnout is common among GPs [9] and the problem seems to be increasing [10]. Burnout is a psychological construct defined as a prolonged response to chronic emotional and interpersonal stressors on the job and is characterized by emotional exhaustion, depersonalization and a subjective experience of decreased personal accomplishment [11]. Burnout has been shown to influence doctors’ ratings of the quality of their own medical care negatively. Thus, burnout has been associated with self-reported suboptimal patient care practices [12], an increased number of self-reported errors among surgeons and primary care physicians [13,14] and self-reported unprofessional conduct and decreased empathy among medical students [15,16]. Thus, the inverse care law may operate in at least three ways: fewer doctors available, less time for appropriate care and impaired doctors due to the effects of increased work pressure.

On this background it is relevant to examine whether GPs working in deprived areas are actually more prone to burnout than GPs working in more affluent areas. We hypothesised that burnout is more frequent among GPs with a high share of disadvantaged patients than among GPs with a low share of disadvantaged patients in the practice population.

The aim of this study was to examine whether burnout in GPs is associated with patients’ socioeconomic characteristics.

## Methods

### Setting

All GPs in Denmark are independent contractors with the regional health authorities and they are fully responsible for the organization of the work in their practice. This also includes premises and staff. According to the national contract, the practice has to be open from 8 AM until 4 PM, from Monday to Friday. Acute patients should be seen the same day and non-acute patients within five weekdays. Virtually all GPs make use of an appointment scheme with consultations of 10–15 minutes. Approx. 40% of GPs are solo GPs. The patient list size is on average 1550 patients per GP (including children) and 99% of citizens are registered with a particular general practice, which they have to consult for medical advice. GPs also act as gatekeepers to the rest of the health care system except for emergencies. GPs are remunerated on a mix of capitation and fee-for-service (25/75%).

### Study population and survey

In January 2012, all 835 active GPs in the Central Denmark Region (1.2 mill inhabitants) were invited to participate in a survey on job satisfaction, burnout, and working conditions (“the GP profile”). The active GPs were identified by the Registry of Health Providers, which is managed by the Regional health authorities. Non-respondents were sent a reminder after four and thirteen weeks and GPs were remunerated in the amount of 50€ for responding. According to Danish law the study was not submitted to an ethical committee since questionnaire surveys do not require an ethical approval. The study was approved by the Danish Data Protection Agency.

### Questionnaire and register-based data

The questionnaire included a burnout scale and items about practice organisation (solo or group practice) and weekly working hours in practice. We used the **Maslach Burnout Inventory Human-Services-Survey** (MBI-HSS), which is considered to be a valid instrument for assessment of burnout symptoms [17]. The scale has been translated into Danish following standardised procedures. The MBI-HSS consists of 22 items and each item is scored on a 7-point Likert scale. The 22 items are divided on three subscales: 1) emotional exhaustion (9 items), 2) depersonalization (5 items), and 3) personal accomplishment (8 items). Each subscale receives a score which is categorised as low or high based on normative population score [17]. A high level of emotional exhaustion is defined as a score >26, and a high level of depersonalization is defined as a score >9. Low personal accomplishment is defined as a score <34 (reverse score). A moderate degree of burnout is defined as a high score on the emotional exhaustion subscale and/or a high score on the depersonalization subscale. A high score on the emotional exhaustion and depersonalization subscales and a low score on the personal accomplishment subscale is defined as a high degree of burnout [17]. Respondents who did not fulfil criteria for either a severe or moderate degree of burnout were classified as not burned-out.

**The Danish Deprivation Index** (DADI) was developed for use in general practice [18]. This index takes a value between 10 and 100 and high numbers indicate more deprived patients in the practice population. The index is calculated in accordance with the Jarman index [19] and the variables and their weights are presented in Table 1. The Danish Regions provided information about which persons were registered with each practice and each patient’s civil registration number. By means of this number, information concerning socioeconomic characteristics was obtained from Statistics Denmark. The socioeconomic characteristics of the practice population were aggregated as proportions per 100 listed persons. We adjusted for net family income using the Oxford equivalence scale suggested by the organization of economic corporation and development (OECD) [20]. A person’s net income was adjusted for household size and composition of adults and children.

**Table 1:** **Variables and weights in the DADI**

**Variable**	**Weight**
Share of 20- to 59-year-old patients, who have been unemployed for more than 6 months	0.100
Share of 20- to 59-year-old patients’ education at high-school level or below	0.125
Share of 20- to 59-year-old patients with low disposable income	0.100
Share of 18- to 59-year-old patients on social benefits	0.100
Share of 0- to 16-year-old children in families with low income	0.150
Share of immigrants and descendants from non-western countries	0.250
Share of patients above 30 years of age living alone	0.075
Share of patients above 70 years of age with a low level of disposable income	0.100
Total	1.000

The DADI was classified as “low”, “medium” or “high” based on the 25^th^ and 75^th^ percentiles: low DADI: ≤ 25^th^ percentile, medium DADI: > 25^th^ to ≤ 75^th^ percentile and high DADI: > 75^th^ percentile.

### Analysis

The difference in mean DADI scores between responding GPs and non-responding GPs was tested with Students unpaired t-test. The scores for burnout were calculated for each responding GP. The associations between burnout and level of DADI and each of the eight DADI variables were calculated as odds ratios (ORs) in separate logistic regression models. We calculated the crude ORs and the adjusted ORs. In the adjusted models, the following potential confounding variables were included: sex and age of GPs and number of consultations during the previous year (2011). As some of the GPs were working in the same practice, the adjusted analyses were corrected for clusters of GPs within the same practice using robust variance estimates. Moreover, analyses were repeated including solo GPs only as a sensitivity test of the results derived from the total population. The 95% confidence intervals (95% CI) for estimates were calculated and p-values of 5% or less were considered statistically significant. Data was analysed using STATA 12.

## Results

In total, 601 (72.0%) GPs returned the questionnaire and 592 had completed the MBI. Among these, 153 (25.5%) reached the criteria for moderate burnout. The characteristics of the study population are shown in Table 2. Calculation of DADI was based on 1474 patients per GP on average (median= 1470, interquartile interval = 1265–1629). The mean DADI for responding and non-responding GPs was 26.3 (SD = 7.1) and 26.9 (SD = 6.4), respectively. This difference was non-significant (t = 1.11; p = 0.266).

**Table 2:** **Characteristics of the participating 601 GPs**

	**All GPs**		**Burned-out GPs**		**Burnout-free GPs**	
	**N = 601 (100%)**		**N = 153 (25.5%)**		**N = 439 (73.0%)**	
	**N**	**%**	**N**	**%**	**N**	**%**
**Sex of GPs**						
Males	312	51.9	77	50.3	232	52.8
Females	284	47.3	74	48.4	204	46.5
Missing information	5	0.8	2	1.3	3	0.7
**Age of GPs**						
34-45	149	24.8	40	26.1	107	24.4
46-53	155	25.8	42	27.5	108	24.6
54-59	154	25.6	40	26.1	113	25.7
60-70	137	22.8	29	19.0	107	24.4
Missing information	6	1.0	2	1.3	4	0.9
Number of consultations during 2011*						
≤ 4363	153	25.5	41	26.8	111	25.3
4364-5187	148	24.6	37	24.2	110	25.1
5188-6008	150	25.0	38	24.8	109	24.8
≥ 6009	150	25.0	37	24.2	109	24.8
**DADI**						
≤ 21.5	154	25.6	35	22.9	114	26.0
21.6-25.5	155	25.8	35	22.9	120	27.3
25.6-30.0	142	23.6	33	21.6	108	24.6
≥ 30.1	143	23.8	50	32.7	90	20.5
Missing information	7	1.2	0	0.0	7	1.6
**Share of unemployed patients (20–59 yrs.)**						
≤1.5%	149	24.8	29	19.0	116	26.4
1.6-1.9%	148	24.6	42	27.5	104	23.7
2.0-2.4%	151	25.1	37	24.2	113	25.7
≥2.5%	146	24.3	45	29.4	99	22.6
Missing information	7	1.2	0	0.0	7	1.6
**Share of patients with education at high-school level or below (20–59 yrs.)**						
≤22.6%	149	24.8	37	24.2	108	24.6
22.7-26.5%	149	24.8	30	19.6	118	26.9
26.6-29.8%	148	24.6	33	21.6	114	26.0
≥29.9%	148	24.6	53	34.6	92	21.0
Missing information	7	1.2	0	0.0	7	1.6
**Share of patients with low disposable income (20–59 yrs.)**						
≤18.2%	149	24.8	31	20.3	115	26.2
18.3-21.9%	148	24.6	33	21.6	114	26.0
22.0-26.6%	150	25.0	50	32.7	97	22.1
≥26.7%	147	24.5	39	25.5	106	24.1
Missing information	7	1.2	0	0.0	7	1.6
Share of patients on social benefits (18–59 yrs.)**						
≤8.5%	150	25.0	28	18.3	118	26.9
8.6-9.8%	148	24.6	34	22.2	114	26.0
9.9-12.0%	148	24.6	39	25.5	107	24.4
≥12.1%	148	24.6	52	34.0	93	21.2
Missing information	7	1.2	0	0.0	7	1.6
**Share of children in families with low income (<17 yrs.)**						
≤6.2%	150	25.0	35	22.9	111	25.3
6.3-8.7%	149	24.8	30	19.6	117	26.7
8.8-11.9%	148	24.6	41	26.8	106	24.1
≥12.0%	147	24.5	47	30.7	98	22.3
Missing information	7	1.2	0	0.0	7	1.6
**Share of immigrants and descendants from non-western countries**						
≤1.2%	149	24.8	32	20.9	114	26.0
1.3-2.5%	148	24.6	41	26.8	105	23.9
2.6-5.4%	149	24.8	45	29.4	103	23.4
≥5.5%	148	24.6	35	22.9	110	25.1
Missing information	7	1.2	0	0.0	7	1.6
**Share of patients living alone (≥ 30 yrs.)**						
≤21.6%	150	25.0	27	17.6	119	27.1
21.7-24.4%	148	24.6	43	28.1	103	23.5
24.5-28.6%	149	24.8	45	29.4	104	23.7
≥28.7%	147	24.5	38	24.8	106	24.1
Missing information	7	1.2	0	0.0	7	1.6
**Share of elderly patients with low disposable income (≥ 70 yrs.)**						
≤19.5%	151	25.1	32	20.9	115	26.2
19.6-24.3%	146	24.3	36	23.5	109	24.8
24.4-30.1%	151	25.1	44	28.8	107	24.4
≥30.2%	146	24.3	41	26.8	101	23.0
Missing information	7	1.2	0	0.0	7	1.6

*Number of consultations per practice in 2011/number of GPs in the practice.

**Excluding patients receiving study grants.

Among GPs with a high DADI, 32.7% were classified as moderately burned-out compared with 22.9% of the GPs with a low DADI. The crude and adjusted ORs for burnout was 1.81 (95% CI = 1.04-3.16, P = 0.037) and 1.91 (95% CI = 1.06-3.44, P = 0.032) for GPs with a high DADI (low DADI as reference group), respectively (Table 3). GPs in the middle DADI quartiles did not differ significantly from GPs with a low DADI in their likelihood of burnout.

**Table 3:** **Summary of logistic regression analyses for variables associated with burnout (N = 592)**

	**Unadjusted**			Adjusted model*		
	**OR**	**95% CI**	**p-value**	**OR**	**95% CI**	**p-value**
**Sex of GPs**						
Males	1.00			1.00		
Females	1.09	0.77-1.56	0.623	0.99	0.68-1.45	0.975
**Age of GPs**						
34-45	1.00			1.00		
46-53	1.04	0.62-1.76	0.882	1.01	0.59-1.70	0.984
54-59	0.95	0.56-1.61	0.839	0.95	0.55-1.63	0.856
60-70	0.73	0.41-1.28	0.265	0.70	0.39-1.27	0.247
**Number of consultations in 2011**						
≤ 4363	1.00			1.00		
4364-5187	0.91	0.52-1.58	0.740	0.87	0.50-1.53	0.634
5188-6008	0.94	0.56-1.59	0.829	0.93	0.54-1.59	0.790
≥ 6009	0.92	0.56-1.52	0.741	0.90	0.54-1.51	0.696
**DADI**						
≤ 21.5	1.00			1.00		
21.6-25.5	0.95	0.53-1.70	0.851	0.98	0.53-1.81	0.940
25.6-30.0	1.00	0.57-1.73	0.986	1.07	0.59-1.94	0.815
≥ 30.1	1.81	1.04-3.16	0.037	1.91	1.06-3.44	0.032
**Share of unemployed patients (20–59 yrs.)**						
≤1.5%	1.00			1.00		
1.6-1.9%	1.62	0.92-2.84	0.096	1.76	1.00-3.10	0.051
2.0-2.4%	1.31	0.73-2.37	0.371	1.49	0.79-2.82	0.220
≥2.5%	1.82	1.03-3.22	0.040	1.98	1.09-3.60	0.026
**Share of patients with education at high-school level or below (20–59 yrs.)**						
≤22.6%	1.00			1.00		
22.7-26.5%	0.74	0.41-1.35	0.330	0.79	0.41-1.50	0.473
26.6-29.8%	0.84	0.48-1.50	0.564	0.97	0.52-1.81	0.921
≥29.9%	1.68	0.94-2.99	0.077	1.93	1.00-3.71	0.049
**Share of patients with low disposable income (20–59 yrs.)**						
≤18.2%	1.00			1.00		
18.3-21.9%	1.07	0.58-2.00	0.822	1.08	0.58-2.02	0.814
22.0-26.6%	1.91	1.11-3.33	0.022	1.94	1.08-3.51	0.027
≥26.7%	1.36	0.76-2.44	0.293	1.35	0.75-2.43	0.314
**Share of patients on social benefits (18–59 yrs.)**						
≤8.5%	1.00			1.00		
8.6-9.8%	1.26	0.67-2.35	0.473	1.35	0.71-2.55	0.362
9.9-12.0%	1.54	0.84-2.80	0.161	1.69	0.91-3.13	0.094
≥12.1%	2.36	1.34-4.14	0.003	2.62	1.45-4.71	0.001
**Share of children in families with low income (<17 yrs.)**						
≤6.2%	1.00			1.00		
6.3-8.7%	0.81	0.44-1.49	0.502	0.91	0.48-1.74	0.776
8.8-11.9%	1.23	0.68-2.20	0.494	1.40	0.75-2.61	0.295
≥12.0%	1.52	0.86-2.70	0.151	1.73	0.93-3.22	0.082
**Share of immigrants and descendants from non-western countries**						
≤1.2%	1.00			1.00		
1.3-2.5%	1.39	0.77-2.52	0.276	1.42	0.77-2.63	0.261
2.6-5.4%	1.56	0.85-2.85	0.153	1.57	0.84-2.92	0.159
≥5.5%	1.13	0.62-2.08	0.686	1.13	0.60-2.13	0.697
**Share of patients living alone (≥ 30 yrs.)**						
≤21.6%	1.00			1.00		
21.7-24.4%	1.84	1.01-3.35	0.046	1.91	1.02-3.58	0.043
24.5-28.6%	1.91	1.08-3.37	0.026	1.98	1.08-3.66	0.028
≥28.7%	1.58	0.88-2.83	0.125	1.64	0.90-3.00	0.108
**Share of elderly patients with low disposable income (≥ 70 yrs.)**						
≤19.5%	1.00			1.00		
19.6-24.3%	1.19	0.65-2.16	0.575	1.25	0.67-2.35	0.483
24.4-30.1%	1.48	0.86-2.55	0.159	1.59	0.90-2.81	0.112
≥30.2%	1.46	0.81-2.63	0.208	1.52	0.81-2.85	0.188

*Adjusted for sex, age and number of consultations and GPs working within the same practice.

Among the eight socio-economic variables included in the DADI, the share of 18-59-year old patients on social benefits was the factor most strongly associated with GP burnout with an adjusted OR of 2.62 (95% CI = 1.45-4.71, P = 0.001) for the highest quartile compared with the lowest (Table 3). Regarding the share of patients above 30 years of age living alone, GPs in the second and third quartiles had increased risk of burnout compared to GPs in the lowest quartile (adjusted OR for second quartile = 1.91, 95% CI = 1.02-3.58, P = 0.043 and adjusted OR for third quartile = 1.98, 95% CI = 1.08-3.66, P = 0.028). The higher risk of burnout in GPs in the highest quartile did not reach statistical significance (adjusted OR = 1.64, 95% CI = 0.90-3.00, P = 0.108) (Table 3).

The sensitivity analysis including 131 solo GPs showed the same tendency that DADI was linearly associated with GP burnout. However, the association was statistically insignificant. Furthermore, the share of 18-59-year old patients on social benefits was the factor with the strongest association with GP burnout (highest quartile vs. lowest quartile: OR = 7.19, 95% CI: 1.57-32.87, P = 0.011).

## Discussion

### Key findings

We found that GPs with a practice population in the highest deprivation (DADI) quartile had nearly twice the risk of being classified as burned-out compared to GPs in the lowest DADI quartile. This was true after adjusting for possible confounders such as sex and age of the GP and number of consultations during the previous year. When examining each of the eight variables included in the DADI individually, a higher share of patients on social benefits was strongly associated with risk of GP burnout.

### Strengths and weaknesses

The high response rate and the use of a validated scale for assessment of burnout strengthen the results of the present study. Moreover, unique Danish registries provided detailed information on the entire patient population in Central Denmark Region. Due to the list-system we knew precisely who were listed with a particular practice at what time. By using this information, a precise socio-economic profile for each practice could be estimated not relying on postcode-based or other neighbourhood-related deprivation measures.

One limitation of the study is that in group practices where GPs share the patient list, it cannot be determined whether all GPs were exposed to the same population. Based on the personal characteristics of the GPs, deprived patients may ask for the GP whom they prefer [21]. However, when repeating the analyses including only solo GPs, the pattern of results was replicated.

The high response rate was prominent taking into account the comprehensive and personal questionnaire. Still, 28% of the invited GPs did not participate. As we do not know whether it was the most or the least burdened GPs who declined to participate, the drop-out may have underestimated as well as overestimated the amount of burnout. Finally, although it has been argued that the MBI subscale scores should be treated as continuous data [17], we applied a categorical approach inspired by previous studies [9,10,22-24]. This could mean that details in the association have been lost, but this should not influence the direction of association and the conclusion. Although burnout is probably expressed along a continuum, the use of normative population-based cut-off scores for caseness of burnout gives an indication of the clinical significance of the findings.

### Comparison with existing literature

Two former studies conducted in the UK revealed evidence of an association between patient deprivation and GP wellbeing [3,4] whereas another UK study did not provide such evidence [25]. The mixed results may be explained by the use of different measures of GP wellbeing as well as deprivation. One of the studies [4] used the Carstairs deprivation score [26] which emphasizes material deprivation (e.g. lack of car ownership and overcrowding). Two studies used the GPs’ postal codes [3,25] whereas one study used the patients’ postal codes [4] for the measurement of deprivation.

A number of factors may explain the associations between patient deprivation and GP burnout. First, deprived patients and especially patients on social benefits have higher levels of multimorbidity and, in comparison to more affluent patients; their quality of life appears to be more negatively affected by the multimorbidity [27,28]. Therefore, GPs with a high share of deprived patients often have to deal with complex health issues, which may increase work pressure. Second, it is a well-known phenomenon that doctors are often recruited from the middle and upper social classes [29]. This can influence the doctor-patient-relation as many diseases have a social gradient affecting first and foremost patients with low socioeconomic position and differences in social class have been shown to be implicated in difficulties of communication [30]. Insofar troublesome communication is more frequent during encounters with deprived patients, this may explain why GPs in deprived areas are more inclined to become burned-out from their work.

Working in a deprived area may be a matter of self-selection with high patient-centred GPs being attracted by the challenges associated with taking care of socially deprived patients. Meanwhile, GPs with a high patient-centred orientation have been shown to find their job more stressful than less patient-centred GPs [31], perhaps as a result of ‘compassion fatigue’ [32]. Thus, the commitment which initially could motivate certain GPs to work in deprived areas might have a boomerang effect. Other self-selection mechanisms may be seen in relation to socio-economic aspects exemplified by our finding that the prevalence of burnout was lower the higher the proportion of non-western immigrants.

A high share of patients above 30 years of age living alone was associated with increased risk of burnout. Literature has consistently identified that unmarried individuals report poorer health and have a higher mortality risk than their married counterparts [33]. Even though the mechanisms responsible for the association between marital status and health are uncovered, the association may explain why GPs with a high share of patients living alone were more burdened by their work.

### Use of a multiple index of deprivation

The main reason for using an index instead of socioeconomic indicators individually is that the indicators are expected to make a broad picture of the different aspects of having a patient population that can produce increased work pressure. However, not all variables have the same impact and we used data weighting inspired by the Jarman Index [19] which was based on GPs’ opinion. According to critics, this approach was problematic since the GPs could only assess the burden on the basis of the patients they were exposed to [8]. A high share of immigrants is traditionally given a high weight, but in the present study, the share of immigrants was not significantly associated with burnout in the total GP population. This suggests that the deriving of weights has to be reconsidered. Even though deprivation indexes may have been developed for the purpose of measuring patients’ needs, the results of this study suggest that the indexes may also be measures of GP workload. Financial incentives directed at GPs with a high score on the deprivation index would make it possible for them to reach the same income target with a shorter list of patients and may be part of the solution when attempting to reduce the increased burnout risk [8]. Other incentives which may be of great importance could be supervision and continuing medical education as also suggested by the results of a former study [34].

The finding that the share of patients on social benefits was more strongly associated with GP burnout than the DADI raises the question about whether a single factor would be a better predictor of workload than composite indexes. However, the denominator for this specific factor was the 18- to 59-year old patients and did not include children and elderly people who are the main users of health services. This study provided insight into the associations with specific variables and thus makes it possible to derive new weights for an index.

## Conclusions

This study revealed a higher prevalence of burnout in GPs with a high share of deprived patients on their list compared to GPs with a low share of deprived patients. The single factor most strongly related to burnout was the share of patients on social benefits. The findings indicate that beside lower supply of GPs in deprived areas, people in these areas may also be served by GPs who are in a condition where they do not perform optimally. The findings of the present study call for intervention in order to overcome the inverse care law and to protect the mental health of GPs working in deprived areas. It would be highly relevant to examine whether supervision, continuing medical education, increased GP:patient ratios and remuneration incentives could reduce the increased risk of burnout associated with high exposure to deprived patients.

## Abbreviations

CI: Confidence interval
DADI: The Danish deprivation index
GP: General practitioner
OR: Odds ratio
